# More than meets the eye: a critical discourse analysis of a Swedish health system reform

**DOI:** 10.1186/s12913-023-10212-4

**Published:** 2023-11-09

**Authors:** Frida Jonsson, Hanna Blåhed, Anna-Karin Hurtig

**Affiliations:** 1 Department of Epidemiology and Global Health, Umeå University, Umeå, Sweden; 2 Arctic Research Center (Arcum) at Umeå University, Umeå, Sweden

**Keywords:** Sweden, Primary health care, Reform, Policy analysis, Discourse, WPR

## Abstract

**Background:**

In line with international trends acknowledging the importance of Primary Health Care (PHC) for improving population health and reducing health inequalities, the Swedish health system is undergoing a restructuring towards the coordinated development of a modern, equitable, accessible, and effective system, with PHC principles and functions at its core. Since discursive analyses of documents underpinning PHC reforms are scarce in Sweden and beyond, the aim of this study was to explore how the reorientation towards *good quality and local health care* has been represented in official government reports.

**Methods:**

Based on a policy-as-discourse analysis, four Swedish Government Official Reports underpinning the *good quality and local health care* reform were interrogated following four questions of Bacchi’s “What’s the Problem Represented to be?” (WPR) approach. By applying the first WPR question, concrete proposals guiding the reorientation were identified, analyzed and thematized into candidate problem representations. These problem representations were then analyzed in relation to previous empirical and conceptual research considering WPR questions two and three, which resulted in the development of three problem representations. Potential silences that the problem representations might produce were then identified by applying WPR question four.

**Results:**

The three problem representations connected the Swedish health system “problem” to a narrow mission, a siloed structure, and a front-line service disconnected, especially from the needs and preferences of individual patients. By representing the problem along these lines, the analysis also illustrated how the policy reorientation towards *good quality and local health care* risk silencing important PHC aspects such as health promotion, equitable access, and human resources.

**Conclusion:**

The results from this study indicate that as discursively framed within concrete proposals, government official reports in Sweden represent the health system problem in particular ways and with these problem representations overlooking several aspects that are central to a health system characterized by PHC principles and functions. In the continued reorientation towards good quality and local health care, these silences might need to be acknowledged.

## Background

Ever since the Declaration of Alma-Ata in 1978 [1], Primary Health Care (PHC) has been deemed central to providing first-contact, continuous, and coordinated health care that is not only universal, accessible, and affordable, but comprehensive and equitable. By constituting a broad approach to health policy and service provision [2], PHC is considered a key pillar of health systems worldwide [3–5]. The importance of PHC to achieve health for all, universal health coverage, and the Sustainable Development Goals was reaffirmed in the 2018 Astana Declaration, which emphasized capacity-building, intersectoral collaboration, and collective action [6]. Against this backdrop, PHC should not be reduced to a level of care or limited to a set of clinical functions (e.g., a family doctor-type service delivered to individuals) [2]. Instead, it should be considered a systemic and population-based approach that integrates, and extends beyond, the organizational activities of “primary care” through concerns with equity, participation, person-centeredness, and the social determinants of health [5, 7]. In this study, the PHC concept is used to capture this focus on principles *and* front-line practices [8, 9].

In a changing international landscape characterized by the acute shocks of COVID-19 and various climate disasters coupled with chronic stressors such as population ageing, workforce shortages, noncommunicable diseases, financial constraints, and technological developments, the expectations of PHC are high in Europe [10–12] and beyond [13, 14]. On the one hand, PHC principles and functions are considered central to improving population health and reducing health inequalities by making health systems more efficient, responsive, and equitable in this changing context [15, 16]. On the other hand, ongoing global trends are simultaneously hindering or hampering many transformative PHC efforts [17]. For example, the integrative, holistic, and empowering emphasis of health promotion is critical to realize the aspirations of PHC [18, 19], but health systems still struggle to reorient in health promoting directions due to various barriers [5, 20]. Human resources also constitute the bedrock of health systems [4, 21], but demographic changes compounded by crises like the COVID-19 pandemic has made it increasingly difficult for countries to recruit, retain and engage a qualified workforce [22]. This notion is further depicted by Dussault and Dubois [23] who discuss how human resource management is often neglected or subject to ad hoc attitudes in health policy.

To strengthen health systems in accordance with PHC principles and functions, concerned actors and institutions need to be directed on what to do, how and why. Such guidance is often formulated in policy, and while this concept may seem straightforward, scholarly controversies exist. While some see it as a formal and rational process where governments objectively respond to preexisting problems, others view it as a discursive practice or a form of language and representation that simultaneously reflects and shapes existing realities [24, 25]. In this later view, policy is not a neutral or logical response to problems but a powerful tool that contributes to creating them while emphasizing some issues and silencing others [26].

### Analyzing policy within the PHC context

Considering the importance of policy in guiding transformative PHC efforts, such processes have been subject to scholarly attention albeit with both understandings of, and approaches to, the policy concept varying. In an article, Shaw ([24], p. 200) describes how ‘rationalist approaches’ that view policy as a “formal government decision” or “process of incremental decision-making” tend to use quantitative or mixed methods to analyze costs and consequences of policies or the complexities of policymaking. From this vantage point, policy analysis either assumes a rational understanding of, and solution to, some fixed problem or involves a pragmatic quest to find problems about which something could or should be done [27]. Either way, a rationalist approach is limited in that it assumes that problems exist outside of the policy process and that the purpose of policies is to provide solutions.

As a critique towards rationalist applications, policy-as-discourse approaches uses qualitative methods to explore how problems are discursively framed or created in proposed solutions [26]. Policy is thereby conceptualized as a process of shifting, diverse and contradictory (in)actions while a policy-as-discourse analysis seeks to uncover hidden assumptions and symbolic meanings through critically examining words, phrases, and narratives [24, 27]. This means that the analysis involves looking into the “items that do make the political agenda to see how the construction or representation of those issues limits what is talked about as possible or desirable, or as impossible or undesirable” ([27], p. 49).

To the best of our knowledge, no research to date has applied policy-as-discourse in the context of PHC. Yet, while this discursive approach has been applied to health-related topics such as palliative care [28], cardiovascular disease [29], mental health [30], and COVID-19 responses [31], the area of PHC has generally been analyzed through more ‘rational’ analyses. Using mixed methods, Gilbert et al. [32] have, for example, investigated the reformation of primary care in Quebec through the creation of family medicine groups. Mosquera et al. [33] have, in turn, conducted quantitative analyses to evaluate effects of the 2010 Swedish free choice reform. By adopting a more critical perspective to PCH inspired changes, Duncan and Reutter [34] illustrate how the policy reorientation towards strengthened home care in Canada seems to be largely situated within a medical and neoliberal paradigm focusing on treatment and efficiency rather than the principles of equity and health promotion. Along the same lines in terms of (in)equities, the New Zealand Government’s PHC strategy of 2001 has been poorly aligned with, and not properly grounded in, the needs, culture, and competencies of the indigenous Māori population [35].

Based on the idea that “what we propose to do about something indicates what we think needs change (‘the problem’)”, through analyzing proposed solutions, policy-as-discourse analyses are important to explicate an often-implicit understanding of what the problem is represented to be ([26], p. xi). Not only do such analyses open specific problem representations up for scrutiny, but they can also help to discover contours and consider limitations to the way problems are formulated. For example, if a policy represents health system problems in particular ways, how could these problem representations have come about and what aspects might they overlook? In this study, we set out to explore these questions within the context of an ongoing reorientation of the Swedish health system towards a more PHC informed *good quality and local health care* [36]*.* This means that we bridge a methodological gap in knowledge since policy-as-discourse analyses have been rarely applied in the PHC context. It also means that we contribute to debates about health system reforms by drawing attention to the potential limits of, and assumptions in, policy which might be important in future assessments of whether proposed solutions align with desired outcomes by addressing the right problems.

## Methods

This policy-as-discourse analysis [24, 26, 27] sets out to analyze a policy aimed at reforming the Swedish health system, focusing specifically on the PHC informed reorientation towards good quality and local health care, through systematically investigating four central government documents. Before describing this material, the context in which the reports have been developed is presented.

### Reforming the Swedish health system towards PHC

Sweden has a decentralized and largely tax-funded governance structure comprising three levels: the central government, the regional councils, and the local councils. As shown in Table 1, there are 21 regional councils and 290 local councils (municipalities) where the former has the responsibility for specialized secondary care and the latter are responsible for various facilities and services. From a PHC perspective, this means that while some principles and functions have a clear division of responsibility (e.g., the regions being responsible for hospitals and the municipalities for school healthcare) other are shared (e.g., primary care and health promotion efforts). As we will see in the results, this structure is somewhat problematic.

**Table 1:** Responsibilities at the different levels

21 regional councils	290 local councils (municipalities)
Specialized health care and dental care	Education, social services, elderly care, childcare, care for people with physical disabilities or psychological disorders and housing, roads, water supply and waste processing
*Shared responsibility* Primary care and health promotion	

Following the principle of local self-government as enshrined in the Swedish Constitution, the regions and municipalities have independent powers of taxation and considerable degree of autonomy to govern public services. This principle also implies that proposals in national documents that guide policy (such as Government Official Reports) may only be advisory to regional and local councils following their right to independent and free self-determination. With reference to health system reforms focusing on PHC, this autonomy can be seen, for example, in 2007 when some regional councils started to reorient health care along with a market-orientated focus on patient choice and free establishment of health centers for private providers [37]. Adherence to this choice-based system then became mandatory for all Swedish regions in 2010 [38], a change that so far seems to have had negative consequences in terms of equity [37, 39].

A few years after the focus shifted towards patient choice and privatization, another broad health system restructuring towards *good quality and local health care* (“God och Nära Vård”) was initiated in 2017 as the central government commissioned an inquiry to support regions, agencies, and organizations in the coordinated development of a modern, equitable, accessible, and effective health system, with primary care at its center [36]. Not only did the focus of this restructuring (e.g., on collaboration and person-centeredness) contrast with the earlier New Public Management inspired free choice reform [37], it represents an ongoing effort to transform the Swedish health system in line with PHC principles and functions. In this study, the four Swedish Government Official Reports [40–43] that contributed to, and resulted from, the commissioned inquiry has been analyzed based in the idea that they constitute “discursive units” ([24], p. 205) central to the *good quality and local health care* reform:

- Effective health care (2016:12) [40].
- Good quality and local health care – a joint road map and vision (2017:53) [41].
- Good quality and local health care – a primary care reform (2018:39) [42].
- Good quality and local health care – a reform for a sustainable health care system (2020:19) [43].

After being published, the government has moved forward with some of the proposals in the reports to make them mandatory rather than advisory for all regional councils. However, since our aim is not to evaluate effects of the *good quality and local health care* reform but to explore how the policy represents problems in particular ways, we have not analyzed this later material.

### The “What’s the Problem Represented to be?” (WPR) approach

This policy-as-discourse analysis was conducted using Bacchi’s [26] “What’s the Problem Represented to be?” (WPR) approach. Influenced by constructionist theories, and with a Foucauldian perspective on problematization [44], WPR is a critical and unconventional approach to policy analysis. Compared to traditionally ‘rational’ analyses [24], the approach is applied to qualitative interrogate policy through texts using a series of pre-determined questions (Table 2). In this regard, distinguishing between a ‘problem’ and a ‘problem representation’ is important. Whilst the problem comprises aspects that need a solution, as in *problem solving* [26], problem representations constitute an illustration of how the problem came to be. This distinction is reflected in WPR questions one to four where the problem representations are first identified by exploring how the problem is represented in reports through solutions proposed (Q1). Otherwise taken-for-granted assumptions (Q2) and non-discursive practices and developments (Q3) contributing to the problem representations are then elicited together with a consideration of the silences that they might produce (Q4).

**Table 2:** Outline of the WPR questions and how they were applied in the analysis

WPR Questions	How the questions were applied in the analysis
1. What is the problem represented to be in reports focusing on *good quality, local health care*?	Proposals were identified through multiple readings of the reports. The proposals were then compiled, analyzed, and thematized into candidate problem representations using Q1
2. What assumptions underlie the representations of the problem?	The candidate problem representations were analyzed in relation to previous empirical and conceptual research, guided by Q2 and Q3. This analytical process resulted in the identified problem representations
3. How have these representations of the problem come about (genealogy)?	
4. What is left unproblematic in these problem representations? Where are the silences? Can the ‘problem’ be thought of differently?	The identified problem representations were scrutinized and discussed in relation to Q4, resulting in the identification of silences that they might produce

Table 2 provides an overview of our analytical process. Following Bacchi’s ([26], p. 3) ideas of “working backwards” by looking into the concrete “items that do make the political agenda” ([27], p. 49), our iterative analytical process was initiated as each author individually read the four reports to identify tangible solutions. We then met to discuss the insights from these initial readings. By applying Q1, all the identified solutions were then compiled, analyzed, and thematized into three candidate problem representations. These candidate problem representations were then considered in relation to Q2 and Q3, which meant that we discussed and analyzed them in relation to previous empirical and conceptual research to understand their underlying assumptions and to trace a few societal developments (i.e., ones that exist outside of the specific policy) that may have contributed to their formation. Following Q1–Q3, this iterative analysis of both readings and writings resulted in the formation of three problem representations. These were then analyzed in relation to Q4, which involved close readings and discussions of the problem representations to identify silences that they might produce.

## Results

By scrutinizing four Swedish Governmental Official Reports guiding the reorientation towards *good quality and local health care* [40–43], three problem representations were developed following Q1–Q3 of the WPR approach [26]: *the narrow mission, the siloed structure, and the disconnected front-line services*. The quotes and information in Tables 3, 4 and 5 have been translated by the authors from Swedish to English and modified slightly for enhanced readability.

**Table 3:** Examples of proposed solutions underpinning the first problem representation

- A road map for the coordinated development of a modern, equitable, accessible, and efficient health system with a focus on primary care needs to be developed
- Regional councils and municipalities must establish an overall joint plan to ensure joint long-term strategic planning for the shared responsibility of front-line services
- It must be investigated how the system needs to change to facilitate the transfer of health care to open forms of care
- The concept of home care must be replaced with the concept of health care in the home to clarify that the care provided in the home is equal to other health care services in terms of required quality

**Table 4:** Examples of proposed solutions underpinning the second problem representation

- A national consultation system consisting of regular meetings at the top political level between the government and all regional councils to strengthen the conditions for more coordinated governance should be introduced
- It must be regulated in the Health and Medical Services Act that where health care activities are carried out, conditions for the collaboration that is needed for good care must be provided. In this way, the requirements for cooperation are also strengthened at the operational level

**Table 5:** Examples of proposed solutions underpinning the third problem representation

- Care must be provided to increase the benefit for the patient and to involve the patient as a co-creator
- The Health and Medical Services Act should include a provision that care must be easily accessible for contact as well as assessment and visits
- The content in the individual plan should be person-centered and the goal should be formulated from the individual’s perspective
- Each patient should have the opportunity to have a patient contract which, in a coherent manner, is based on the individual's needs and preferences

### The narrow mission

*There is a need for a shared goal in the transition from today’s hospital-centered health system to a new front-line health system. A health system with primary care as its basis, that builds upon interactions between hospitals and municipal initiatives, and that clearly responds to patients’ needs. (*[41]*, p. 75)*

As the quote illustrates and as detailed in Table 3, this first problem representation was developed from proposed solutions concerning the need to establish a shared goal, a roadmap, and a plan for change towards a front-line health system that has its basis in the principles and functions of PHC. Located at a more conceptual, systems level, this problem representation also builds on solutions (Table 3) calling for further inquiries and clarity in concepts to ensure a more comprehensive front-line services, thus representing the problem as a “narrow mission” (Q1).

When attempting to “trace the history” of the first problem representation (Q3) ([26], p. 10), our analysis suggests that it may be the result of and contingent upon decades of investment in specialized secondary care in Sweden. Not only does front-line services currently receive only about 16% of regional budgets [45], but the health system as a whole has been largely governed since the 1980–90 s through the competition, control and cost containment of NPM [46]. This means that the general and comprehensive front-line services have been comparatively under-resourced, fragmented and inequitably developed [37, 47] despite ambitions to increase resource allocation, promote interorganizational collaboration and implement a more “trust-based” governance system [48]. Representing the problem as a narrow mission by proposing solutions to transition towards a more needs-based, integrated, and comprehensive health system may also be a reaction to the widespread ideals and assumptions of the medical prestige hierarchy and the curative “disease model” (Q2). Because by emphasizing dominant biomedical and technological perspectives, such approaches tend to contribute to the concentration of status and resources (both human and financial) in the realms of secondary care rather than front-line services [5, 49].

As discussed above, the first problem representation may be a response to the negative consequences of various health-system ideals and prioritizations in Sweden favoring narrow, disease-centered specializations over more comprehensive, people-centered generalizations (Q3). In relation to these developments, the problem representation may also emerge from evidence about the benefits of PHC principles and functions for improved health outcomes and service delivery [15, 16], through an increased focus, for example, on prevention, promotion, and rehabilitation (Q2). However, while the value of PHC has received widespread international attention ever since 1978 with the Declaration of Alma-Ata [1], this focus has been largely concentrated on low- and middle-income countries. This means that discussions about PHC have generally been less visible in high-income countries such as Sweden [50, 51], which may have contributed to the development of “today’s hospital-centered health system” (Q3) ([41], p. 75).

### The siloed structure

*Based on the municipalities’ increased commitment as principal [providers] of health care [in addition to the regional councils] since the law in force was formulated, collaboration between the principals must be strengthened. (...) In order to make the requirements for collaboration clear, the collaborative responsibilities of regions and municipalities in the planning and development of health care need to be clarified. (*[43]*, p. 160)*

The second problem representation was developed from proposed solutions calling for improved steering from national to local levels, in addition to those drawing attention to the collaborative roles and responsibilities of the regional councils and the municipalities, as illustrated in the quote and detailed in Table 4. Specifically, by emphasizing the need for collaboration and coordination when patients require care from both principal providers, the problem is located at an organizational systems level by being represented as a “siloed structure” (Q1).

Building on international [4, 5] and Swedish [47] PHC literature, this problem representation could be a result of and emerge from widespread ideas about the benefits of integrated health systems, for example, when it comes to access, quality, and continuity (Q2). While the concept of integration has many inherent elements, it is usually a response to complex and “wicked” problems that require practices and partnerships to extend across traditional administrative boundaries [52]. However, the value of integrated care for managing emerging challenges such as noncommunicable diseases typically relies not only upon integration *within* health systems but also upon the integration that takes place *across* health and social care services [53]. As such, building on solutions calling for enhanced collaboration and coordination, especially when patients require care from regional and municipal providers, this problem representation may also emerge from a recognized value of intersectionality for care provision in general, and the realization of PHC principles and functions in particular [54].

Building on the decentralized governance structure and widespread introduction of NPM, this problem representation should also be seen in relation to the fragmentation and increased marketization of the Swedish health system (Q3), involving “a [policy] shift from an egalitarian towards a libertarian ideology” ([51], p. 3). Within this context and as detailed in Table 1, the 21 regional councils and the 290 municipalities share the responsibility for primary care, which until now has contributed to a somewhat differentiated care organization with unclear mandates. By promoting intersectoral collaboration between otherwise largely autonomous actors, the problem representation may also emerge from a desire to improve health system efficiency, effectiveness, and accountability through preventing and managing fragmentation [4, 55] (Q2).

### The disconnected front-line service

*Primary care should provide expert information, advice, and support based on the individual needs and preferences of patients who themselves, or with the help of relatives, can take measures to improve their situation. (*[42]*, p. 335)*

The final problem representation was developed from proposed solutions concerned with the provision of front-line services, focusing particularly on approaches that would contribute to improved accessibility and the integration of patients’ needs and preferences in service delivery, as illustrated in the quote and detailed in Table 5. In this regard, the problem is located at a micro system level by being represented as a “disconnected front-line service” (Q1).

Following from solutions concerned with the relevance of services for individual patients—their needs and preferences—this problem representation may be contingent upon pervasive PHC discourses and developments calling for more person-centered models of care [e.g., 5, 12] (Q3). As discussed in the literature [46, 56], approaches emphasizing the influence and involvement of patients in the design and delivery of care (e.g., through voice, choice, and co-production) are not new, but have come to permeate much of health policy and practice nationally and internationally during the last decades. While the motive behind concerns with person-centeredness can be ethical (e.g., respect for people’s autonomy has an intrinsic value), Nolte, Merkur and Anell [56] discuss how its application has also become increasingly instrumental (i.e., as a means to other beneficial ends). Against this backdrop, the problem of a ‘disconnected front-line service’ may emerge from ideas that a more person-centered model has the potential to improve care quality and outcomes (Q2) [12, 57].

This problem representation as underpinned by solutions calling for more person-centered models of care may also be a response to the limiting discourses and practices of medical paternalism characterized by top-down power relationships between patients and health care professionals (Q2) [58]. Alternatively, it may be a reaction to the increased bureaucratization of the Swedish health-sector governance since the 1980s and the increasingly widespread implementation of NPM since the 1990s (Q3) [46]. Beyond contributing to a focus on efficiency and productivity, the paternalism and bureaucracy of NPM typically make patients objects of, rather than subjects in, their care. As such, the problem of a ‘disconnected front-line service’ may emerge from changes in a Swedish health system that has become permeated by a market-oriented focus and seemingly less able to care for patients with more complex needs [51].

## Discussion

The aim of this study was to explore how problems are represented in the ongoing reorientation towards a more PHC informed *good quality and local health care* in Sweden by conducting a policy-as-discourse analysis following the WPR approach [24, 27]. As well as providing a structure for scrutinizing representations of problems in the reports, Bacchi [27] directs attention towards issues that are silenced in problem representations. By considering the “limits” of the above outlined problem representations by asking “what fails to be problematized”? ([26], p. 12), below we discuss three potential silences considering previous research. The first one follows from a disregard with the role of prevention and health promotion. The second builds on the lack of focus on health care access, and the third emerges from the scarce attention paid to human resources.

### Silence around prevention and health promotion

PHC principles and functions play key roles in building comprehensive health systems that improve population health and reduce health inequalities [15, 16]. In this context, “comprehensive” implies providing services that treat, cure, and rehabilitate patients who are or have been sick, but also broader attempts focused on preventing disease and promoting health [18, 19]. As part of many transformative PHC efforts internationally, research and reports have emphasized the importance of strengthening health systems through prevention and health promotion [4, 5]. By representing the problem as a narrow mission (in the sense of prioritization having so far been focused mainly on specialized secondary care), the first problem representation recognizes the centrality of front-line services, but simultaneously overlooks their role and responsibility for ensuring that people remain healthy. Since none of the other problem representations drew attention to this issue, there seems to be a silence around the need for disease prevention and health promotion within the health system more generally and front-line services in particular.

### Silence around equitable health care access

Accessing health care is a right, not a privilege, which makes health care access paramount to the democratic governance of a welfare state [59]. In line with the principles of PHC [3, 4], the Swedish Health and Medical Services Act [60] stipulates that health care should be equitable and easily accessible based on need. However, during the last few decades, access has been constrained, partially due to the “Achilles’ heel” of widespread prolonged waiting times [61]. Barriers to accessing health care have become especially apparent for groups marginalized by factors such as low socioeconomic position [62], migrant background [63, 64], or living in remote rural areas [39, 65]. To improve access, scholars have stressed the need to align the characteristics and expectations of (potential) patients with the services provided by through concepts such as approachability, acceptability, availability, affordability, and appropriateness [66]. By locating the problem primarily *within* the health system as it exists and is organized today, we suggest that the above problem representations also risk silencing the needs of people and populations who—for various social, cultural, economic, or geographical reasons—cannot easily access services as they are currently designed and delivered.

### Silence around human resources

The health care workforce constitutes a cornerstone of any health system [21]. Compared to most OECD countries, Sweden allocates large amounts of human resources to the health sector, while having a highly skilled cadre of providers [67]. However, considering population ageing and other demographic changes, recruiting and retaining workers constitutes an existing and future challenge, for both the health system and society at large [22]. By locating the problem primarily in relation to organizational siloes and limited patient-centeredness, the problem representations seem to also disregard the central health-system pillar that is human resources [4]. Specifically, by representing the problem as discussed above, the value and voices of health workers might be overlooked which, during times of reformation and other pressures on health systems, are so desperately needed [21].

## Conclusions

Against the backdrop of international transitions towards PHC to strengthen health systems, improve population health and reduce health inequalities [10–14], discursive analyses of documents guiding these developments are important to shed light on potential limits of, and assumptions in, the policy processes [26]. By explicating an often-implicit understanding of what the problem is represented to be, such analyses may also contribute knowledge useful in evaluations of whether policy proposals have led to desired or expected outcomes by addressing the right problems.

In the current study, we conducted a policy-as-discourse analysis to contribute such knowledge by exploring how problems are represented in the ongoing reorientation towards a more PHC informed *good quality and local health care* in Sweden. We also considered how the problem representations may have come about, and what aspects they might overlook. Specifically, by following Bacchi’s WPR questions one to three [26], we identified and developed three problem representations. These represent the Swedish health system ‘problem’ as (i) the mission being narrow, (ii) the structure being siloed, and (iii) the front-line services being disconnected, especially from the needs and preferences of individual patients. By considering these problem representations in relation to potential silences that they might produce [26], we suggest that central PHC aspects such as health promotion, equitable access, and human resources [4, 21] may be overlooked.

In the continued Swedish health system reform, the findings from this research might be worth considering in policymaking and practice to achieve a good quality and local health care. An international audience might, in turn, be prompted to explore ongoing PHC developments critically and discursively by delving deeper into the policy-as-discourse approach [24]. Despite this, we acknowledge that discursive analyses, such as ours, are limited in that they draw attention to the ways policy is shaped by language and representation while overlooking other important aspects. We also recognize that while we have analyzed a rich and relevant set of documents, the results might have been different if other or additional sources of data had been interrogated. Additionally, our study is limited in that we only discuss three silences that seemed especially important considering the PHC literature when there are many more aspects that failed to be problematized in the problem representations.

## Data Availability

All data generated or analyzed during this study are included in this published article.

## Abbreviations

NPM: New Public Management
PHC: Primary Health Care
OECD: Organization for Economic Cooperation and Development
WPR: “What’s the Problem Represented to be?
